# The effects of erector spinae plane block on perioperative opioid consumption and rehabilitation in video assisted thoracic surgery

**DOI:** 10.1186/s12871-021-01536-x

**Published:** 2021-12-10

**Authors:** Sen Zhang, Xiaodan Han, Di Zhou, Minli Sun, Jing Cang, Changhong Miao, Chao Liang

**Affiliations:** Department of Anesthesiology, Zhongshan Hospital, Fudan University, Shanghai, 200032 China

**Keywords:** Erector spinae plane block, Thoracic epidural anesthesia, Video-assisted thoracic surgery, Opioid consumption, Postoperative rehabilitation

## Abstract

**Background:**

This study aimed to determine whether ultrasound-guided continuous erector spinae plane block (ESPB) had an effect on opioid consumption and postoperative rehabilitation in patients undergoing video-assisted thoracic surgery (VATS).

**Methods:**

In this prospective study, 120 patients aged 20–70 years who underwent elective VATS were randomly allocated to one of three groups: group C (general anesthesia with patient-controlled intravenous analgesia [PCIA]), group T (general anesthesia with patient-controlled epidural analgesia [PCEA]), or group E (general anesthesia with continuous ESPB and PCIA). Perioperative opioid consumption, visual analog scale (VAS) scores, preoperative and postoperative Quality of Recovery-15 scores, and postoperative opioid-related adverse events were all assessed.

**Results:**

Intraoperative sufentanil consumption in groups T and E was significantly lower than that in group C (both *P < 0.001*), and the postoperative sufentanil consumption in group E was also significantly lower than that in group C (*P = 0.001*). Compared with group C, the VAS scores at rest or during coughing immediately out of the post-anesthesia care unit at 6 h, 12 h, and 24 h postoperatively were significantly lower in group T (*P* < 0.05). However, the VAS scores at rest at 6 h and 12 h postoperatively in group E were lower than those of group C (*P* < 0.05), but were significantly higher than those of group T at all study times (*P* < 0.05).

**Conclusion:**

Ultrasound-guided continuous ESPB significantly reduced perioperative opioid consumption during VATS and improved postoperative rehabilitation. However, these effects were inferior to those of thoracic epidural anesthesia.

**Trial registration:**

The present study was prospectively registered at http://www.chictr.org/cn /(registration number: ChiCTR1900023050); registration date: May 82,019.

## Introduction

Over the past decade, video-assisted thoracoscopic surgery (VATS) has become the most widely used surgical technique for managing primary lung cancer [1]. Compared with thoracotomy, VATS is associated with a shorter convalescence period, less pain, and better survival rates [2]. However, some patients still experience moderate to severe acute pain after VATS, particularly within 24 h postoperatively [3–5].

There are many modalities to alleviate post-thoracic surgical pain, ranging from various medications for patient-controlled analgesia to diverse regional analgesic methods [6]. Thoracic epidural anesthesia (TEA) remains the “gold standard” for intraoperative analgesia and management of acute post-thoracic surgical pain [7, 8]. In terms of pain relief, thoracic paravertebral block (PVB) is comparable to TEA, which is widely applied in thoracic surgery [9]. However, TEA is more invasive and can lead to devastating complications, and cannot be used in patients with severe spinal deformities who are receiving anticoagulation treatment [10–13]. In addition, PVB is not widely used because it requires multiple injections and carries a risk for complications [14].

In 2016, Forero et al. described the erector spinae plane block (ESPB), which is a new technique for indirect PVB methods [15]. Since then, many studies have reported that ESPB is safe and easy to use. A recent study showed that preoperative ESPB may offer an equivalent quality of recovery and analgesia after VATS as compared with PVB [16]. In ESPB, local anesthetics are injected into the fascial plane, deep into the erector spinae muscle, which is distant from the pleura and neuraxial structures. Through drugs penetrating the intertransverse connective tissues, ESPB not only affects the ventral rami and dorsal side of the spinal nerve in the paravertebral space, but also the lateral cutaneous branches of the intercostal nerves [14, 15].

It has been widely reported that ESPB can provide effective regional thoraco-abdominal analgesia during cardiothoracic surgery, breast surgery, or laparoscopic cholecystectomy [17, 18], as well as lengthen the duration of regional anesthesia. A block can be administered continually with the help of a catheter, which can provide better postoperative analgesia and can be an alternative to TEA for pain management [19–21]. However, these data are mainly from case reports; there is a paucity of research on randomized post-VATS ESPB studies. Therefore, prospective and randomized studies comparing the benefits of ESPB, traditional anesthesia, and other analgesic regimens such as general anesthesia with or without TEA, are needed. Moreover, other debilitating side effects aside from pain may also affect the patient’s recovery experience, and it remains unclear whether ESPB could improve postoperative rehabilitation.

The present study was designed to determine whether ultrasound-guided continuous ESPB has an effect on opioid consumption and postoperative rehabilitation as compared with general anesthesia with or without TEA.

## Methods

This study was approved by the Institutional Review Board of Zhongshan Hospital, Fudan University (B2019-074R) and written informed consent was obtained from all individuals participating in the trial. The trial was registered prospectively prior to patient enrollment at http://www.chictr.org/cn/ (registration number: ChiCTR1900023050, Principal investigator: Chao Liang, date of registration: 08/05/2019). The study protocol was performed in accordance with the relevant guidelines and has been reported in line with the guidelines of the Consolidated Standards of Reporting Trials.

### Study population

Patients aged 20–70 years, with an American Society of Anesthesiologists physical status (ASA PS) of 1 or 2 and a diagnosis of solitary pulmonary nodules without chronic pain or with no pain medications routinely used were deemed suitable to undergo 3-port single-intercostal VATS, as performed by surgeons. The exclusion criteria were pre-existing infection at the block site, history of chronic pain, significant coagulopathy, contraindication to techniques or drugs used in the protocol, and conversion to open thoracotomy.

### Randomization and patient grouping

According to a computer-generated randomization list, patients were assigned to one of three blocks, with a sealed envelope technique, to one of three groups: group C (general anesthesia with patient-controlled intravenous analgesia [PCIA]), group T (general anesthesia with patient-controlled epidural analgesia [PCEA]), or group E (general anesthesia with continuous ESPB and PCIA).

### Method of anesthesia and analgesia

On arrival at the operating room, routine monitoring, including invasive blood pressure, pulse oxygen saturation (SpO_2_), and electrocardiography were performed. In group T, the patients were placed in a left lateral decubitus position, and a thoracic epidural catheter (19G; Pajunk GmbH Medizintechnologie, Germany) was inserted at the thoracic (T) T7 to T8 epidural space by an experienced anesthesiologist before induction. In group E, the patients were placed in a left lateral decubitus position before induction, and a high-frequency linear ultrasound transducer was placed in a longitudinal orientation, 3 cm lateral to the T5 spinous process. Three muscles superficial to the hyperechoic transverse process shadow were identified as follows: trapezius, rhomboid major, and erector spinae. Under ultrasound guidance, an 8-cm, 22-gauge block needle was inserted in-plane in a caudad-to-cephalad direction, until the tip was laid on the surface of the transverse process. The correct needle tip position was confirmed by visualizing the linear fluid spread that separated the erector spinae muscle from the transverse process. Then, 30 mL of 0.375% ropivacaine (AstraZeneca AB) was injected deep into the erector spinae muscle, and a thoracic epidural catheter was subsequently inserted. After confirmation and assessment of the sensory block to pinprick, induction of general anesthesia was initiated.

General anesthesia was induced with propofol (Corden Pharma S.P.A) target-controlled infusion (TCI) (target plasma concentration was set at 4.0 μg/ml), remifentanil (Jiangsu Nhwa Pharmaceutical Co., Ltd) (0.2 μg/kg/min), sufentanil (Yichang Renfu Pharmaceutical Co. Ltd) (0.2 μg/kg), and rocuronium bromide (0.6 mg/kg). Patients were intubated using a double-lumen tube to achieve lung isolation; correct positioning was confirmed using fibreoptic bronchoscopy. After induction, ropivacaine (0.1875%, 5 mL) was injected into the epidural space of the patients in group T every 5 min for a total of three times; ropivacaine (0.1875%, 5 mL) was injected into the epidural space every hour during surgery. One-lung ventilation was initiated when the operation was started. Anesthesia was maintained with sevoflurane (Shanghai Hengrui Pharmaceutical Co., Ltd.) (0.8 MAC). During the surgical procedure, 5 μg of sufentanil was administered intravenously to both groups for maintaining systolic blood pressure changes within 20% of the baseline. This dose was repeated every 10 min until the blood pressure returned to the required limits. Rocuronium was administered as required.

All patients in the three groups were administered the same electronic analgesia pump (AM380; ACE Medical Co. Ltd., Gyeoggi, Korea). In group C, the drugs used for PCIA were sufentanil (1 μg/kg) and ramosetron (Chongqing Lummy Pharmaceutical Co., Ltd.) (0.6 mg), which were diluted in 0.9% normal saline to a final volume of 250 mL. The analgesia pump settings were as follows: background dose, 0 mL/h; self-controlled additional dose, 4 mL/time; and lockout time, 6 min. In group T, the drugs administered for PCEA were ropivacaine (0.12%) and sufentanil (0.6%), diluted in 0.9% normal saline to a final volume of 250 mL. The analgesia pump settings were as follows: background dose, 3 mL/h; self-controlled additional dose, 4 mL/time; and lockout time, 10 min. In group E, the drugs administered for continuous ESPB analgesia were ropivacaine (0.2%), diluted in 0.9% normal saline to a final volume of 250 mL. The analgesia pump settings were as follows: background dose, 7 mL/h; self-controlled additional dose, 0 mL/time; and lockout time, 40 min. A PCIA pump, with the same settings as for group C, was also used in group E to evaluate postoperative sufentanil consumption.

The intraoperative and postoperative sufentanil consumption in each group was recorded. During the preoperative preparation, patients were instructed to evaluate their pain using the following: visual analog scale (VAS), with scores ranging from 0 to 10 (0 = no pain, 10 = worst pain); and VAS scores at rest and during coughing immediately out of the post-anesthesia care unit (PACU) at 6 h, 12 h, and 24 h postoperatively. Before the day of surgery, the investigators asked patients to complete the Quality of Recovery-15 (QoR-15) questionnaire as a measure of baseline (relatively healthy) status. They were then asked to repeat the questionnaire 24 h postoperatively. Opioid-related adverse events, such as nausea, vomiting, dizziness, hypotension, pruritus, and respiratory symptoms, were also recorded.

### Statistical analysis

The primary endpoint of this study was intraoperative sufentanil consumption. The secondary endpoints were the following: postoperative sufentanil consumption; VAS scores at rest and during coughing immediately out of the PACU at 6 h, 12 h, and 24 h postoperatively; QoR-15 at 24 h pre- and postoperatively; and postoperative opioid-related adverse events.

Normality testing was conducted using the Kolmogorov–Smirnov test. All data are reported as mean (standard deviation [SD]), median (inter-quartile range), or number (percentage), as appropriate. Normally distributed continuous variables were compared using a one-way analysis of variance (ANOVA). Non-normally distributed continuous variables were compared using the non-parametric Kruskal–Wallis test. Categorical variables were analyzed using the chi-square test and Fisher’s exact test. All data were processed using IBM SPSS Statistics 21.0 (IBM Inc., New York, NY). Statistical significance was defined as a two-sided *P*-value < 0.05.

In a pilot study of 45 patients, the mean (SD) intraoperative sufentanil consumption was 38.0 (9.8), 23.0 (6.0), and 25.3 (6.0) in groups C, T, and E, respectively. A sample size of 31 participants in each group was calculated using one-way ANOVA to show a 20% difference in the mean intraoperative sufentanil consumption for an expected SD of 10, with a statistical power of 90% and an alpha error level of 0.05. To allow for attrition, the sample size was increased to 120.

## Results

**Fig. 1:**
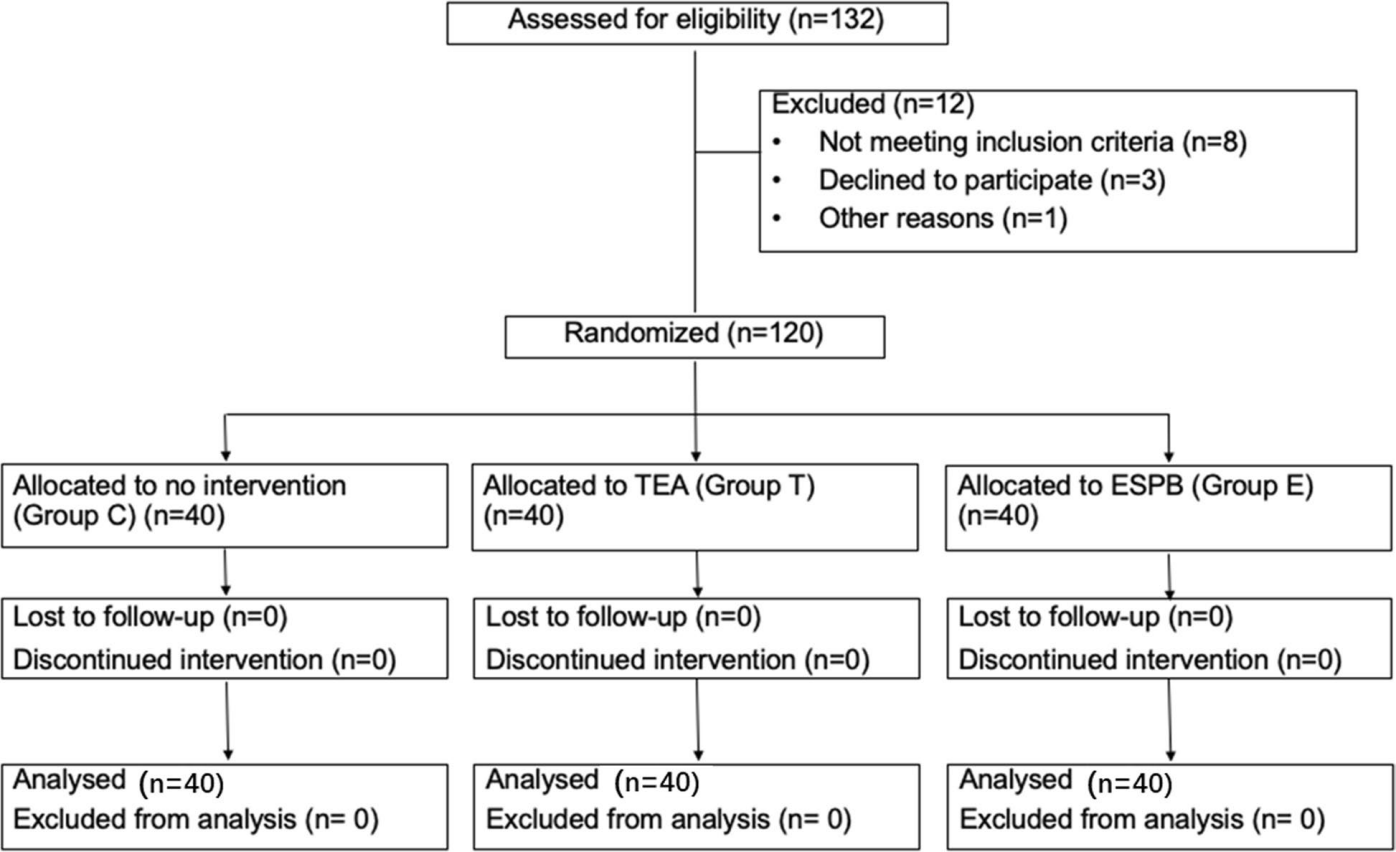
CONSORT flowchart. While 132 patients were initially screened as potentially suitable, 120 patients were finally randomized and included in the study. Group C, general anesthesia with patient-controlled intravenous analgesia (PCIA); group T, general anesthesia with patient-controlled epidural analgesia (PCEA); and group E, general anesthesia with continuous ESPB and PCIA. CONSORT, Consolidated Standards of Reporting Trials; ESPB, erector spinae plane block

A total of 120 patients participated in this study. Forty participants were randomly assigned to each group (Fig. 1). Both patient and surgical characteristics are shown in Table 1.

**Table 1:** Subject and surgical characteristics

	Group C	Group T	Group E
	(*n* = 40)	(*n* = 40)	(*n* = 40)
Age (yr)	54.3 (11.9)	55.4 (10.4)	54.3 (13.6)
Gender (M/F)	15/25	12/28	12/28
Weight (kg)	62 (9.6)	62.8 (10.5)	59.7 (12.2)
Height (cm)	164.3 (8)	164.2 (7.4)	163.9 (8.1)
BMI (kg/m^2^)	22.8 (2.4)	23.2 (3.0)	22.1 (3.2)
ASA PS (I/II)	22/18	18/22	26/14
Duration of surgery (min)	80 (26.2)	87.1 (27.9)	84.9 (34.8)
Surgical procedures (n [%])			
Wedge resection	11 (27.5)	11 (27.5)	9 (22.5)
Segmentectomy	11 (27.5)	9 (22.5)	12 (30)
Lobectomy	18 (45)	20 (50)	19 (47.5)

Data are expressed as mean (standard deviation). Group C, General anaesthesia with patient-controlled intravenous analgesia (PCIA);Group T, General anaesthesia with patient-controlled epidural analgesia (PCEA); Group E, General anaesthesia with continuous ESPB and PCIA. *BMI *Body mass index, *ASA PS *American Society of Anesthesiologists physical status

**Fig. 2:**
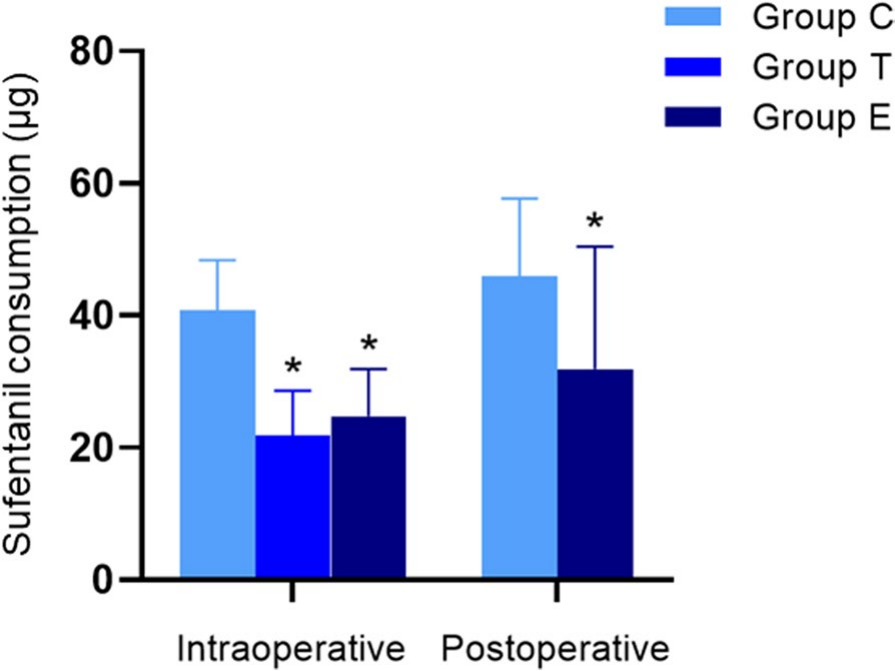
Perioperative sufentanil consumption. Group C, general anesthesia with patient-controlled intravenous analgesia (PCIA); group T, general anesthesia with patient-controlled epidural analgesia (PCEA); and group E, general anesthesia with continuous ESPB and PCIA. CONSORT, Consolidated Standards of Reporting Trials. ^*^P < 0.05 versus group C

**Fig. 3:**
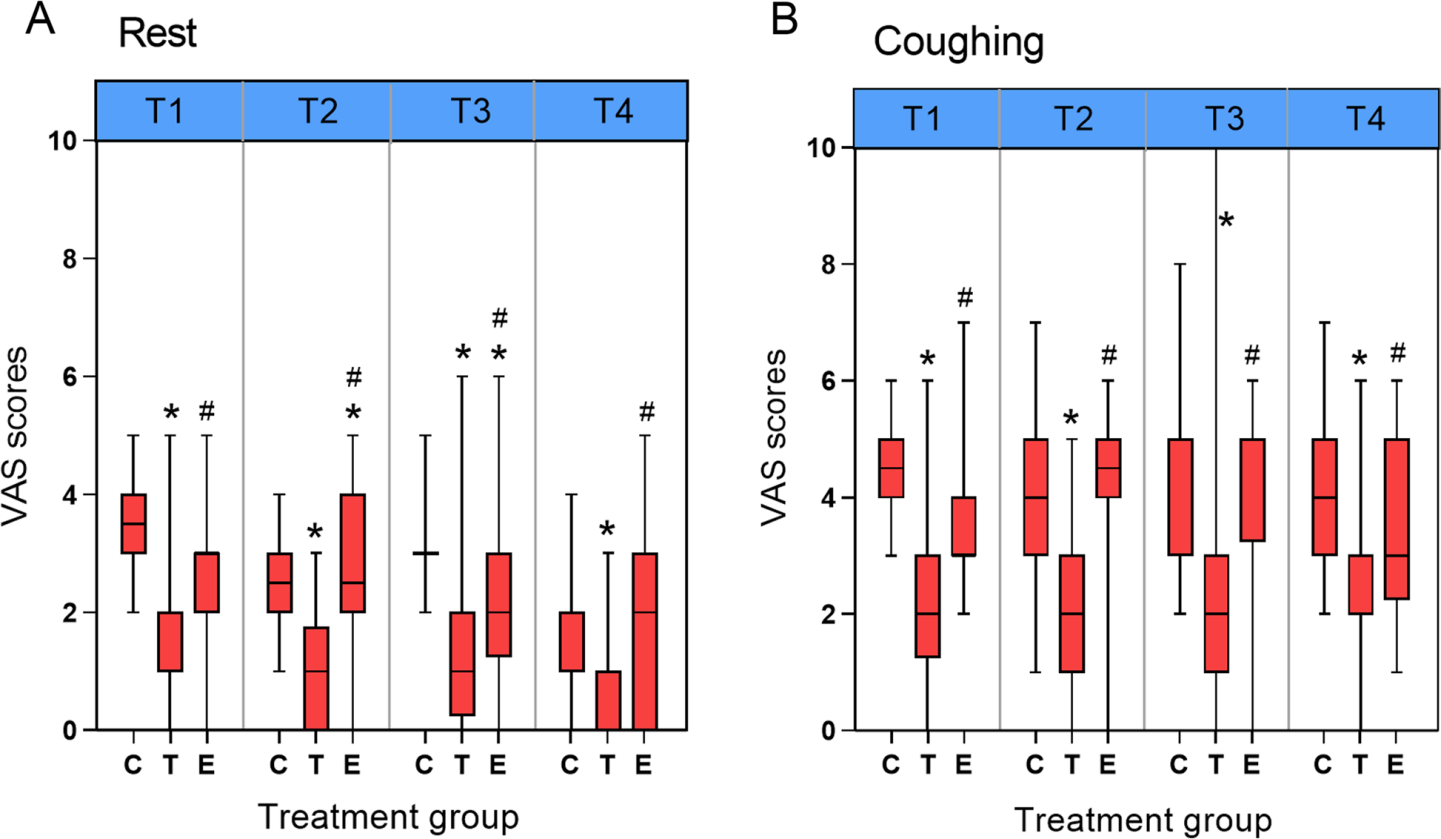
Box plot of scores for the VAS by study groups across different study times: T1 = immediately out of post-anesthesia care unit (PACU); T2 = postoperative 6 h; T3 = postoperative 12 h; T4 = postoperative 24 h. Group C, general anesthesia with patient-controlled intravenous analgesia (PCIA); group T, general anesthesia with patient-controlled epidural analgesia (PCE); and group E, general anesthesia with continuous ESPB and PCIA. ^*^P < 0.05 versus group C. ^#^*P* < 0.05 versus group T. Median values shown as solid line. The whiskers represent the 5th and 95th percentile values. (A) VAS scores at rest; (B) VAS scores during coughing. VAS, visual analog scale

The intraoperative sufentanil consumption in groups T and E was significantly lower than that in group C (both *P* < 0.001), and no significant differences in intraoperative sufentanil consumption were found between groups T and E. Moreover, the postoperative sufentanil consumption in group E was also significantly lower than that in group C (*P* = 0.001) (Fig. 2). Compared with group C, the VAS scores at rest or during coughing, across different study times, were all significantly lower in group T (*P* < 0.05) (Fig. 3). However, the VAS scores in group E were lower than those in group C only at rest at 6 h and 12 h postoperatively (*P* < 0.05). Compared with group T, the VAS scores of group E were significantly higher at all time points (*P* < 0.05) (Fig. 3).

**Fig. 4:**
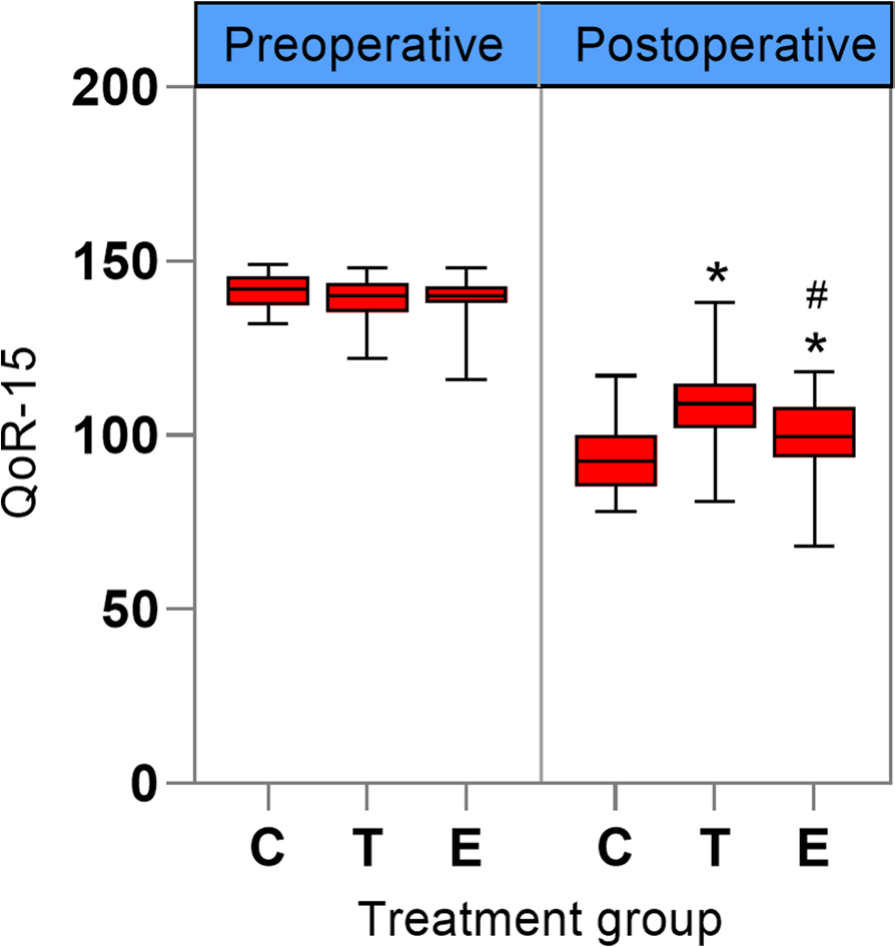
Box plot of preoperative and postoperative scores for the QoR-15. Group C, general anesthesia with patient-controlled intravenous analgesia (PCIA); group T, general anesthesia with patient-controlled epidural analgesia (PCEA); and group E, general anesthesia with continuous ESPB and PCIA. ^*^P < 0.05 versus group C. ^#^*P* < 0.05 versus group T. QoR-15, Quality of Recovery-15

The preoperative baseline values of QoR-15 were comparable between the two groups, while the postoperative QoR-15 values of groups T and E were significantly higher than those of group C (*P* < 0.001 and *P =* 0.004, respectively); however, the postoperative QoR-15 value in group E was lower than that in group T (*P =* 0.0005) (Fig. 4). The incidence of postoperative nausea and vomiting was lower in group E than in groups C and T, but the difference was not statistically significant (both *P =* 0.154). In addition, TEA significantly increased the incidence of pruritus compared to groups C and E (both *P =* 0.005) (Table 2).

**Table 2:** Postoperative opioid-related adverse events

	Group C	Group T	Group E
	(*n* = 40)	(*n* = 40)	(*n* = 40)
Nausea and vomiting	7 (17.5)	7 (17.5)	2 (5)
Dizzy	5 (12.5)	7 (17.5)	4 (10)
Hypotension	0 (0)	0 (0)	0 (0)
Pruritus	0 (0) ^a^	8 (20)	0 (0) ^a^
Respiratory depression	0 (0)	0 (0)	0 (0)

Data are shown as n (%). ^a^*P* < 0.05 versus Group T. Group C, General anaesthesia with patient-controlled intravenous analgesia (PCIA);Group T, General anaesthesia with patient-controlled epidural analgesia (PCEA); Group E, General anaesthesia with continuous ESPB and PCIA

## Discussion

Many reports have demonstrated effective analgesia using ESPB for the management of postoperative pain in patients undergoing VATS [22]; however, few studies have comprehensively compared the efficacy of ESPB with traditional anesthesia and other analgesic regimens. Herein, we investigated whether ultrasound-guided continuous ESPB had an effect on opioid consumption and postoperative rehabilitation. The results showed that, as compared with general anesthesia with PCIA, continuous ESPB significantly reduced perioperative opioid consumption and improved postoperative rehabilitation in patients undergoing VATS. However, the analgesic and rehabilitation improvement effects of ESPB were inferior to those provided by TEA.

Using ultrasound, regional nerve blocks can be performed precisely with minimal risk. Therefore, there has been a resurgence of interest in nerve blocks that were once considered difficult to perform, such as paravertebral block, which has been demonstrated to have similar efficacies as with epidural analgesia [23, 24]. As a novel technique that may have the potential to supplement the current modalities used for analgesia [15], ESPB can cause somatic, visceral, and sympathetic nerve block at multiple levels and may improve analgesia and lung function after VATS. However, our results showed that although continuous ESPB provided better analgesia than PCIA postoperatively, the average VAS score in group E was higher than that in group T, which indicated that the effects of continuous ESPB for postoperative analgesia were inferior to those of continuous TEA. This may be due to the limited penetration of local anesthetics from the fascial plane into the pleural and neuraxial structures. In our study, after a single shot for ESPB, instead of an intermittent bolus, a continuous infusion regimen of local anesthetics was implemented. Therefore, an effective pressure gradient between the injected fascial plane and the lamina of the thoracic vertebrae could not be established, which significantly affected the postoperative analgesic effects of continuous ESPB. This may explain why the VAS scores in group E were lower than those of group C at rest only at 6 h and 12 h postoperatively, but not at 24 h postoperatively. Thus, the analgesic effects in group E might have been mainly produced by the first single shot of local anesthetics before anesthesia induction. This speculation was also supported by the evidence that the time for the first required analgesia was 6–7 h postoperatively in patients with ESPB undergoing VATS [25]. Therefore, applying an intermittent bolus protocol in ESPB for postoperative analgesia was more suitable [21]. However, the superiority of each administration regimen remains unclear [26]. A recent pooled review of all published studies regarding ESPB reported 80% single-shot techniques, followed by continuous infusions (8%) and intermittent boluses (12%) [22]. Further studies are needed before a more reasonable administration regimen is determined.

Recent studies have compared ESPB and serratus anterior plane block for the management of postoperative pain following VATS [25, 27]. In these studies, the primary outcomes were as follows: pain severity, time for first postoperative analgesia requirement, and intraoperative and postoperative analgesic requirements. A trigger point was set for anesthesiologists to intervene with analgesia in the postoperative period, with a VRS score of > 2 or 4 as the threshold. However, we only calculated the total opioid consumption, since each patient in groups E and C received a PCIA analgesic regimen with a background dose of the PCIA pump set at 0 mL/h. Additionally, all patients in groups C and E were well educated preoperatively on how to correct the PCIA. Moreover, in our pilot study, we found that patients who received PCEA had excellent analgesic effects; thus, we did not apply an additional PCIA pump in patients in group E.

Although reduction of pain is important, it may not be perceived by the patient as a better recovery experience if they experience other debilitating side effects. The QoR-15 is a multidimensional, patient-reported instrument used for functional recovery assessment [28]. The main domains of QoR-15 include pain, physical comfort, physical independence, and psychological and emotional states. The questions of these domains use a 10-point scale ranging from 0 to 10, with reversed scoring for negative questions, and the sum of the individual domains generates the global score (0, worst recovery; 150, optimal recovery). A previous study reported that ESPB can provide superior quality of recovery at 24 h, better analgesia, and lower morbidity after minimally invasive thoracic surgery [27]. Another recent study also revealed that as a part of multimodal analgesia, ESPB has a potential for enhanced recovery from VATS [29]. For a more accurate evaluation of patient recovery, baseline QoR-15 values were collected for all enrolled patients. Given the patient factors such as fatigue and anxiety related to impending surgery, the ability of QoR-15 in the immediate preoperative period to provide an accurate baseline has been questioned [30]. However, no significant differences were found between the groups in the present study. Compared with general anesthesia with PCIA, the postoperative QoR-15 value was significantly higher in patients who received continuous TEA and ESPB analgesia. Since a change in the score of 8 or more signifies a clinically important improvement or deterioration, the data from the present study may reaffirm the important role played by regional analgesia in improving postoperative rehabilitation after VATS. However, the postoperative QoR-15 value of group E was lower than that of group T, which may indicate that the rehabilitation improvement effects of ESPB are inferior to those provided by TEA.

In the present study, a lower incidence of PONV was found in the TEA analgesia group, which may indicate lower opioid consumption and a lower incidence of PONV. However, the higher incidence of pruritus in the TEA analgesia group than in the ESPB and PCIA groups may be attributed to the epidural use of sufentanil, which was consistent with the results of previous studies [31, 32]. The patients in the TEA analgesia group showed no hypotension postoperatively in present study. This might because patients in our study were relatively young and healthy, and awake patients were educated to use the analgesia pump when the pain was obvious; furthermore, our study might have a pretty limited sample size, so more work needs to be done to verify and confirm the results.

The present study had some limitations. First, we investigated the analgesic effects of ESPB on three-port VATS; however, one- and two-port VATS were also prevalent in these years. Therefore, a larger study involving more types of VATS is needed to investigate the analgesic effects of ESPB on VATS. However, recently published expert opinions suggest that pain levels are similar to those of patients who undergo VATS [27]. Second, our study did not investigate the incidence of postoperative complications, such as pneumonia, surgical site infection, and acute kidney injury. It has been reported that regional anesthesia may be associated with a lower incidence of these complications [27]. Third, we only collected analgesia and rehabilitation information until 24 h postoperatively, since acute postoperative pain is a powerful predictor of post-thoracotomy pain syndrome (PTPS) [33]. In a future study, we plan to investigate the effects of continuous ESPB on long-term pain, such as at 48 h or 72 h postoperatively, and on the incidence of PTPS. Fourth, patients in our study were relatively young and healthy, which might limit the applicability of our results to other thoracic surgery patient groups.

## Conclusion

In conclusion, compared to general anesthesia with PCIA, general anesthesia combined with continuous ESPB resulted in a dramatic reduction in opioid consumption in VATS. Moreover, the ESPB improved postoperative rehabilitation. However, the analgesic effects and improvement of rehabilitation due to ESPB were inferior to those provided by TEA. These findings may provide some information or insights for future clinical studies in this area.

## Data Availability

Reasonable requests for access to the datasets used and/or analysed during this study can be made to the corresponding author.

## Abbreviations

ESPB: Erector spinae plane block
VATS: Video-assisted thoracic surgery
PCIA: Patient-controlled intravenous analgesia
PCEA: Patient-controlled epidural analgesia,
TEA: Thoracic epidural anaesthesia
PVB: Thoracic paravertebral block
SpO_2_: Pulse oxygen saturation
TCI: Target controlled infusion
VAS: Visual analog scale
PACU: Post-anaesthesia care unit
QoR-15: Quality of Recovery-15
ANOVA: One-way analysis of variance
